# Supplementary material for: Selective analysis of cancer-cell intrinsic transcriptional traits defines novel clinically relevant subtypes of colorectal cancer
Source: Nat Commun. 2017 May 31;8:15107. doi: 10.1038/ncomms15107 (PMC5499209; doi:10.1038/ncomms15107)
Supplement: Supplementary Information — Supplementary figures and supplementary tables. [file ncomms15107-s1.pdf]

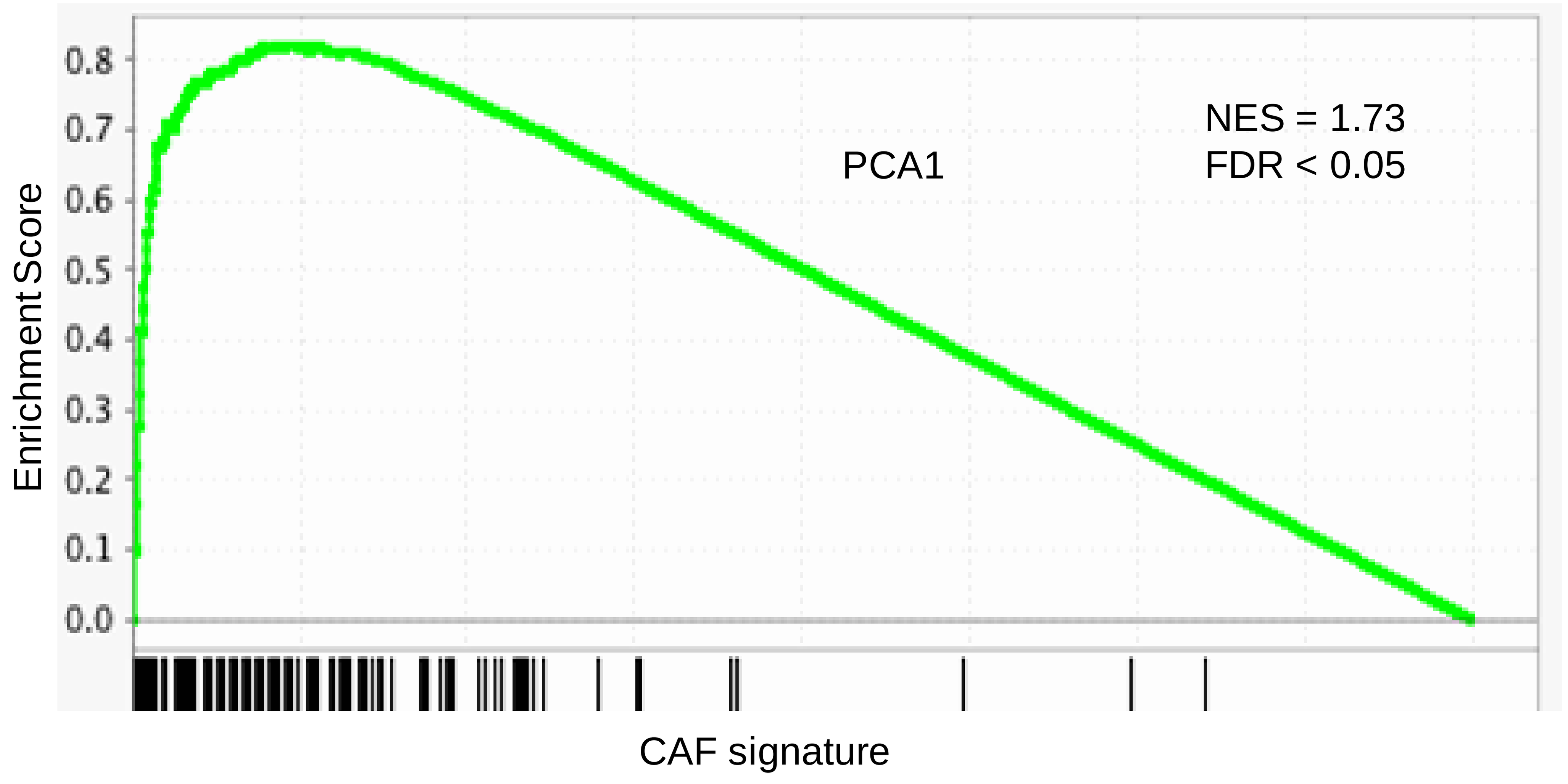

Supplementary Figure 1. Stromal-derived genes are a major determinant of the CRC transcriptome.
Preranked Gene Set Enrichment Analysis of a signature of cancer-associated fibroblasts (Isella et al., *Nat. Genet.* 2015) on the first principal component of the TCGA gene expression dataset.

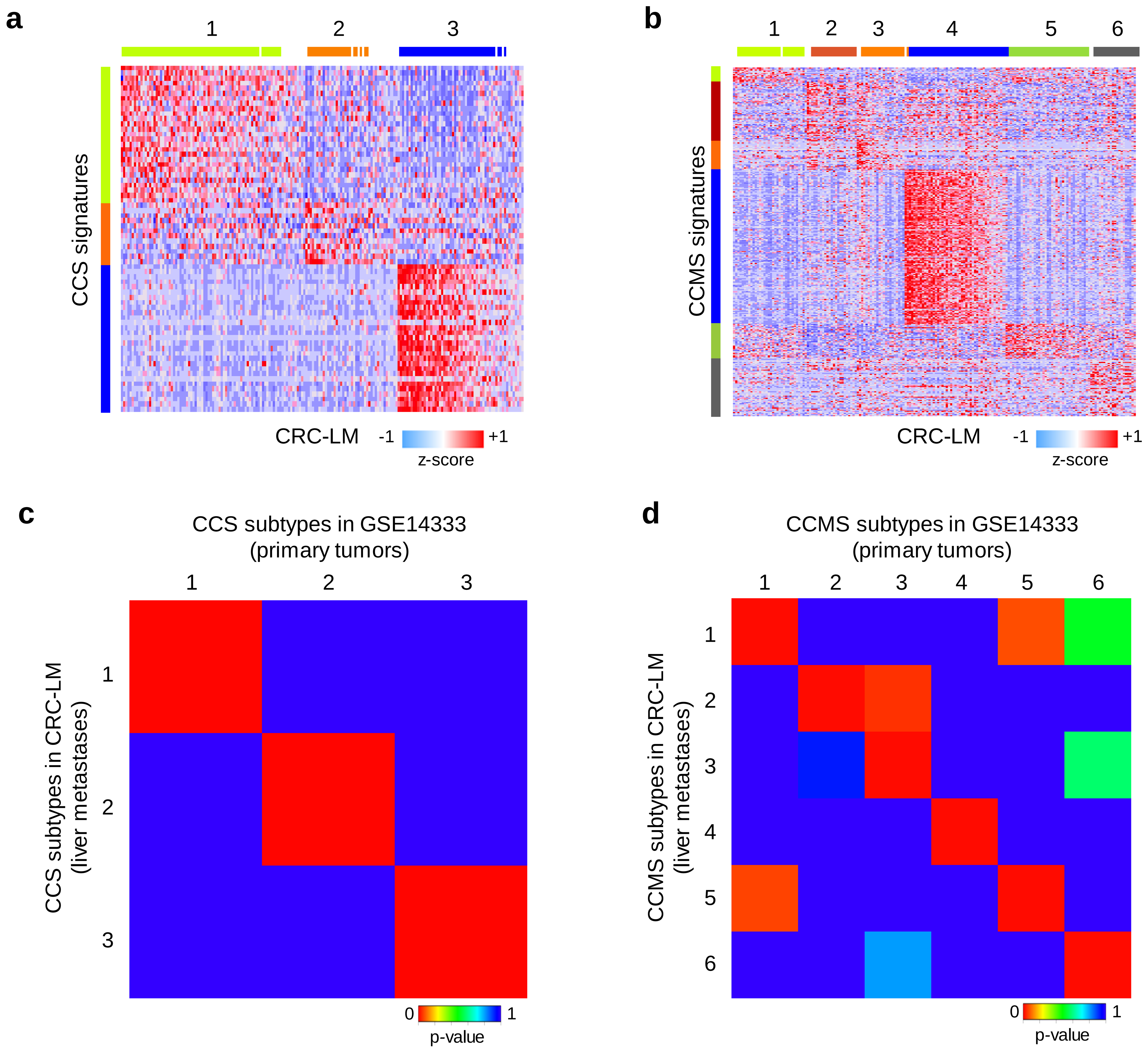

Supplementary Figure 2. Subtyping of CRC liver metastases by CCS and CCMS classifiers.
(**a**, **b**) Heatmap showing NTP classification of the CRC-LM dataset based on the signatures of the CCS (**a**) and CCMS (**b**) classification systems. (**c**, **d**) Submap analysis of CCS (**c**) and CCMS (**d**) subtype similarity between CRC liver metastases (CRC-LM) and primary tumors (GSE14333). P values were calculated by Fisher's exact test using the Submap tool available from Gene Pattern.

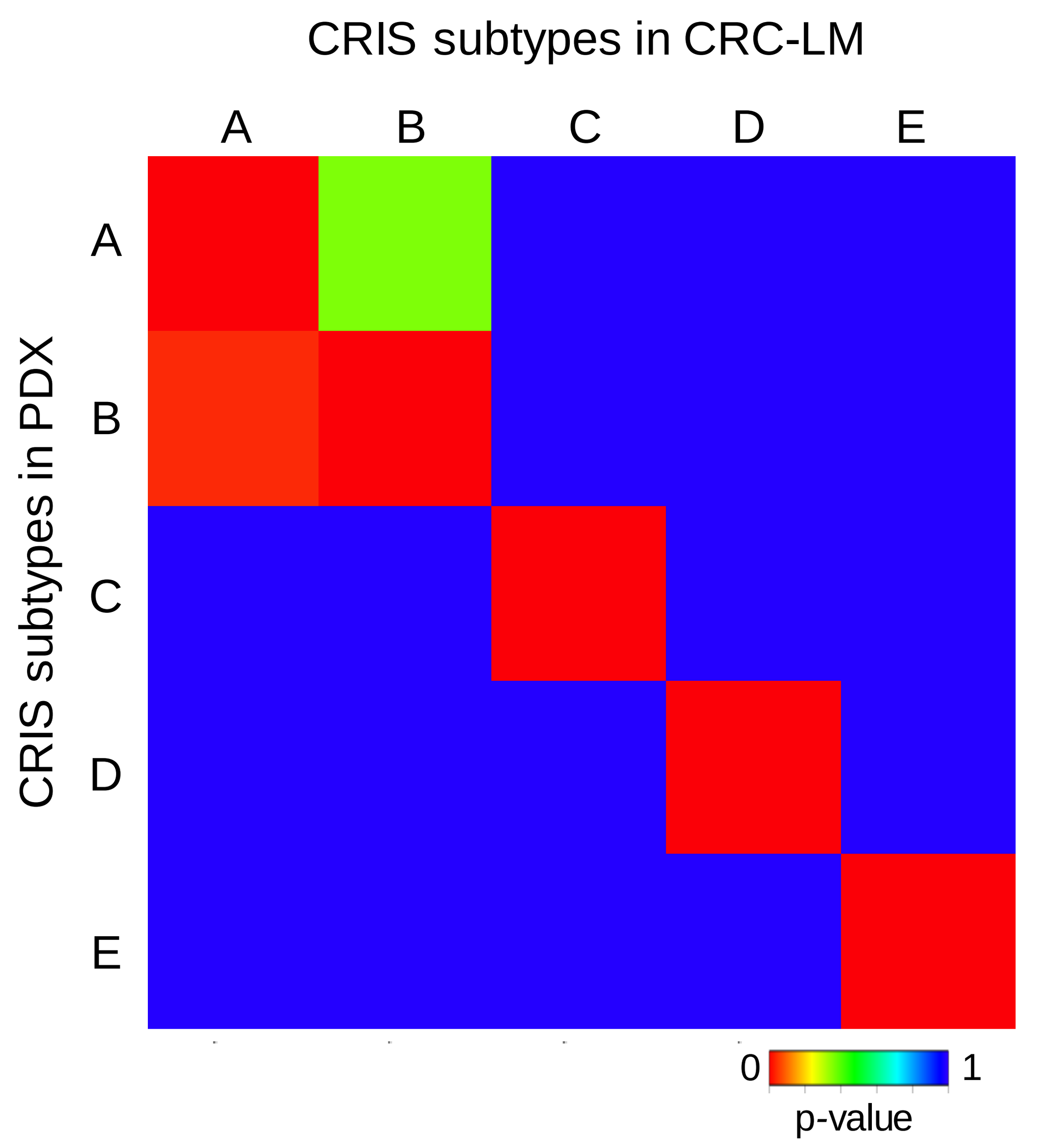

Supplementary Figure 3. Comparison of the transcriptional traits underlying CRIS subtypes in PDXs and in their corresponding original tumors from the CRC-LM dataset.
Submap analysis of CRIS subtype similarity between PDXs and their corresponding original counterparts. P values were calculated by Fisher's exact test using the Submap tool available from Gene Pattern.

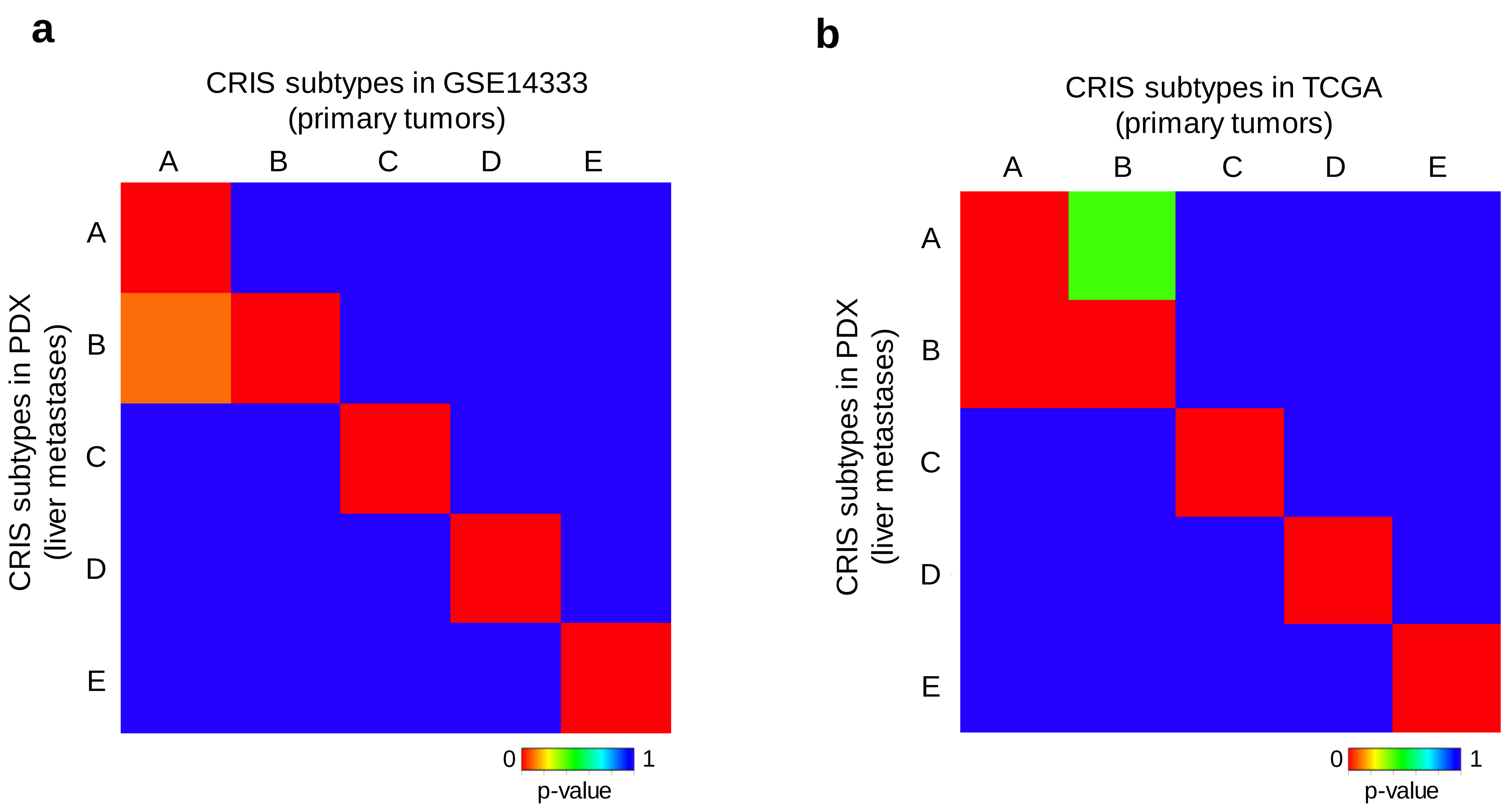

Supplementary Figure 4. Comparison of the transcriptional traits underlying CRIS subtypes in PDXs of CRC liver metastases and in primary CRC tumors.
Submap analysis of CRCA subtype similarity between PDXs of CRC liver metastases and primary tumors from GSE14333 (Affymetrix microarrays) (**a**) and TCGA (RNAseq) (**b**). P values were calculated by Fisher's exact test using the Submap tool available from Gene Pattern.

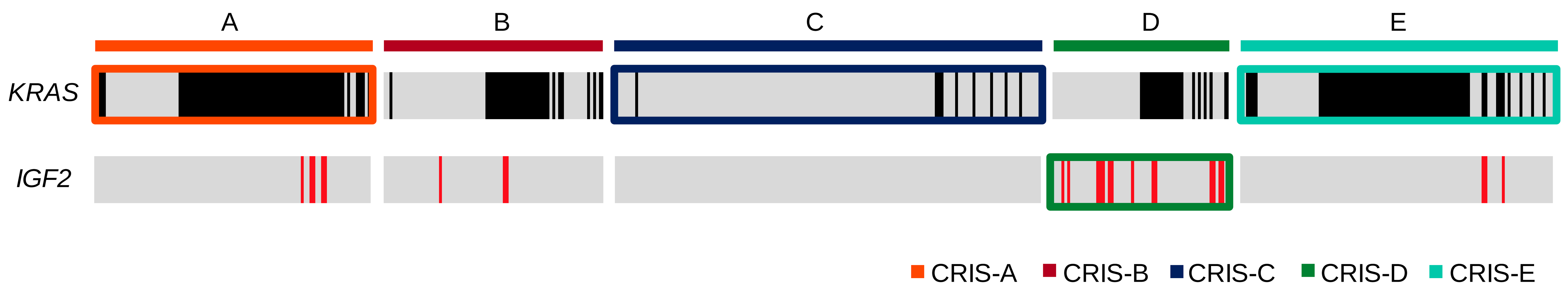

Supplementary Figure 5. Genetic features of CRIS subtypes in the PDX dataset.
Distribution of *KRAS* sequence alterations and *IGF2* overexpression (defined as z score > 2) in the PDX dataset. Significant subtype enrichments are evidenced by colored boxes. Fisher's exact test, $P < 1x10^{-8}$ for mutant *KRAS* enrichment in CRIS-A; $P < 1x10^{-12}$ for mutant *KRAS* depletion in CRIS-C; $P < 5x10^{-3}$ for mutant *KRAS* enrichment in CRIS-E; $P < 5x10^{-4}$ for enrichment of *IGF2* overexpression in CRIS-D.

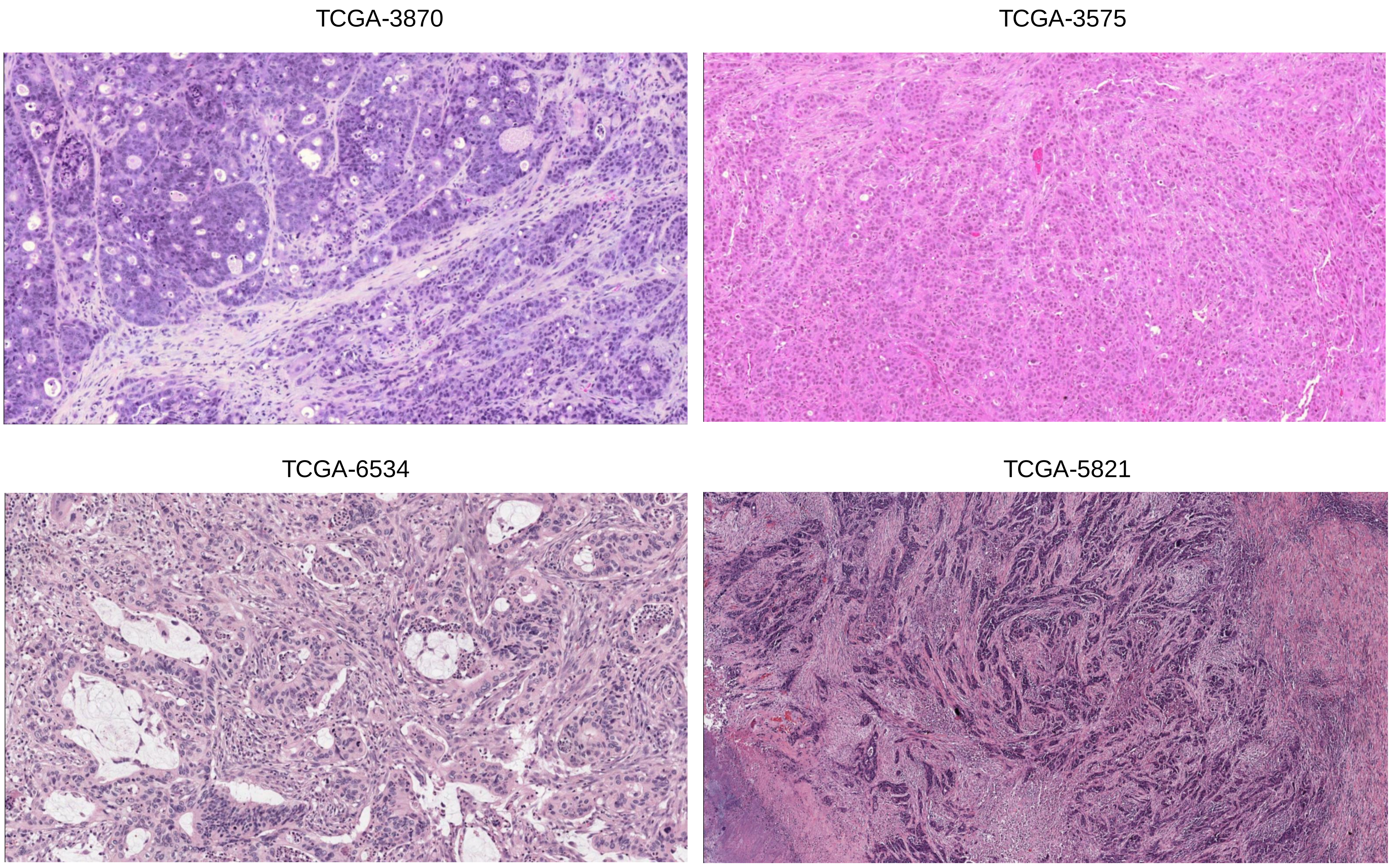

Supplementary Figure 6. Histological features of CRIS-B tumors in the TCGA dataset.
Hematoxylin-eosin staining of slides from four poorly differentiated tumors, representative of CRIS-B histology. Glandular structures constitute less than 50% of the tumor and are substituted by sheets and nests of cancer cells. Images were obtained from http://cancer.digitalslidearchive.net (Gutman et al., *J. Am. Med. Inform. Assoc.* 2013).

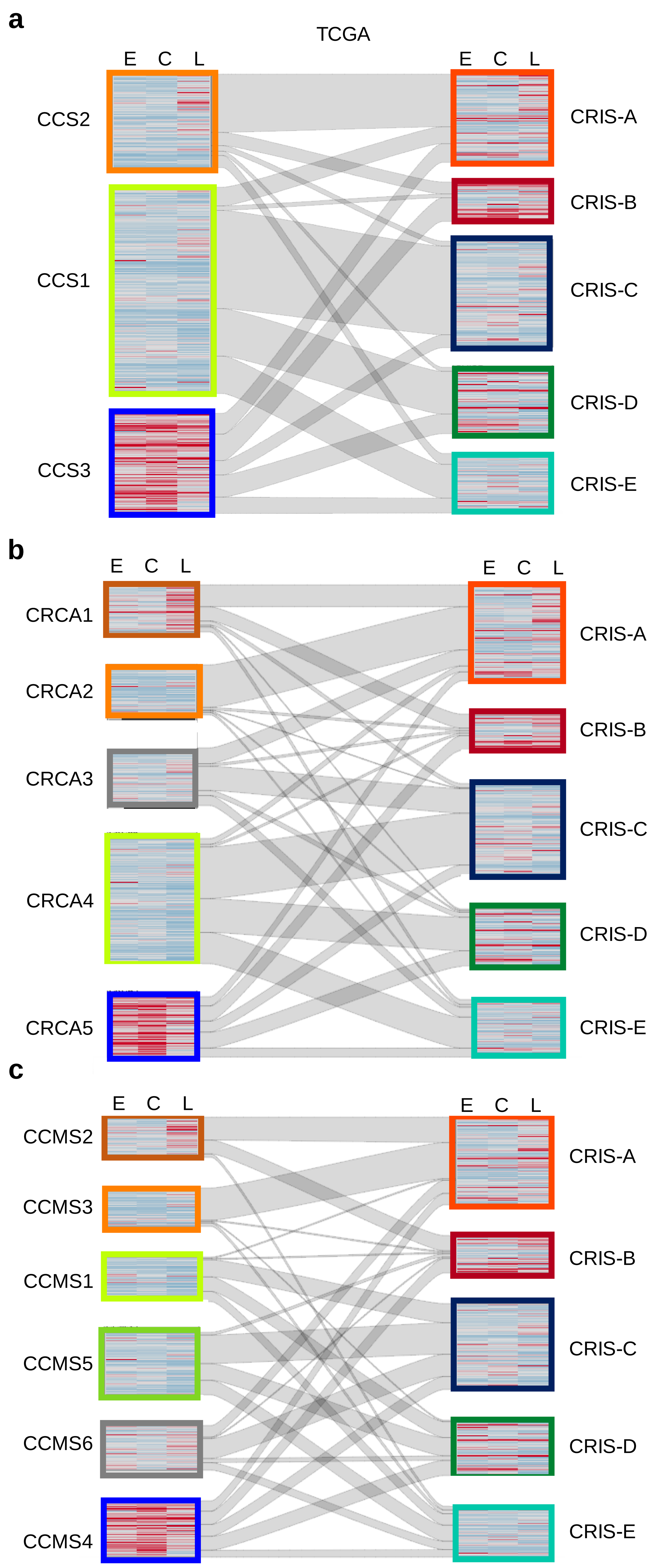

Supplementary Figure 7. Comparison between CRIS classes and three public CRC classifiers. Caleydo view of correspondences between CRIS classes and CCS (**a**), CRCA (**b**) or CCMS (**c**) subtypes in the TCGA dataset. Heatmaps show estimates of stromal infiltration derived from gene expression analysis of specific signatures (E, endothelial cells; C, CAFs; L, leukocytes).

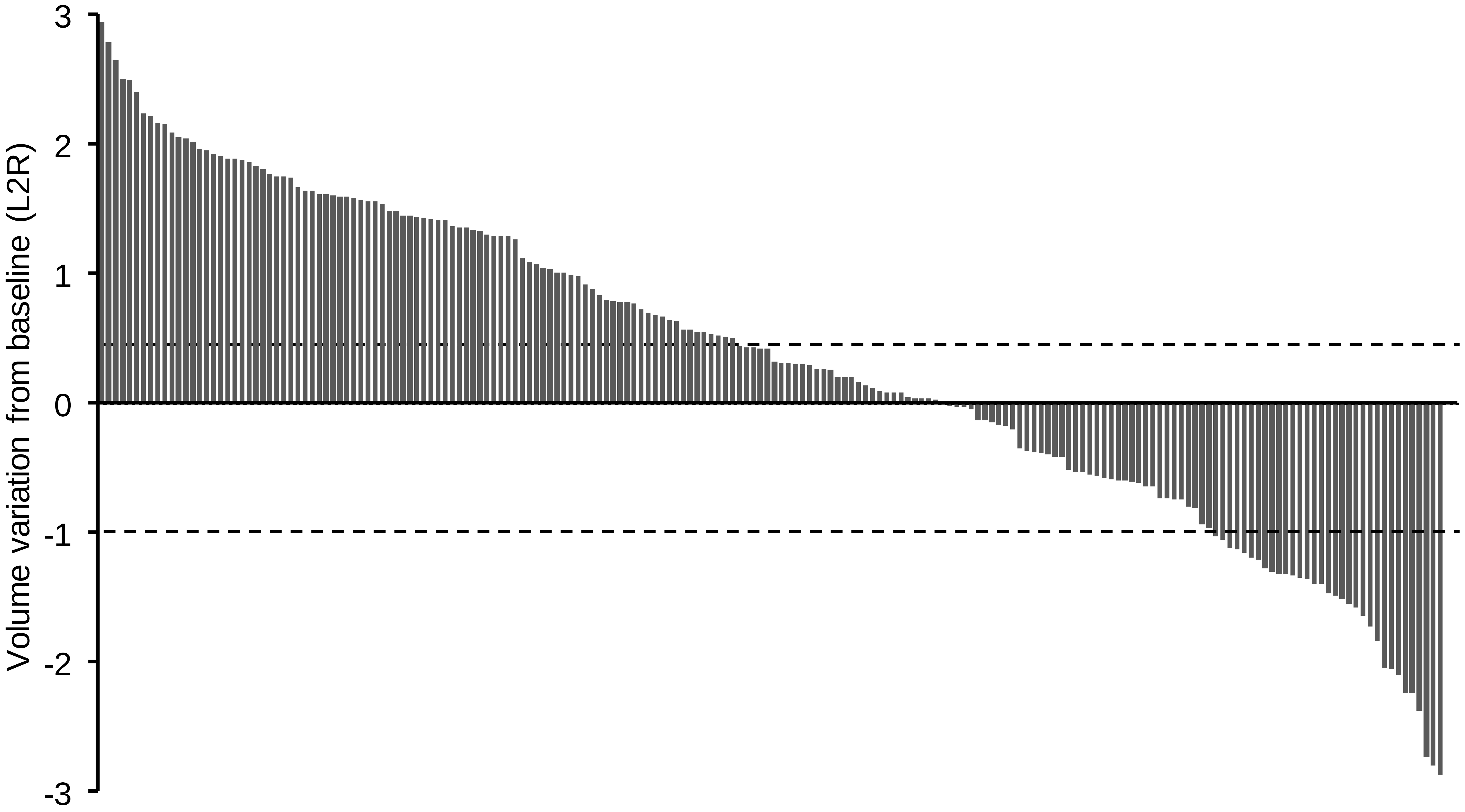

Supplementary Figure 8. Response to cetuximab in PDX cohorts.
Waterfall plot of response to cetuximab (20 mg/kg, twice a week) after three weeks of treatment, compared with tumor volume at baseline, in a population of 192 PDX cases. The dotted line indicates the cut-off value between resistant cases (progressive disease, arbitrarily defined as a tumor volume increase of at least 35%) and sensitive cases (a combination of stable diseases, with tumor volume changes between 35% increase and 50% reduction, and objective responses, with tumor volume reduction of at least 50%). Each bar denotes the average growth change in treatment cohorts of 6 or 12 mice.

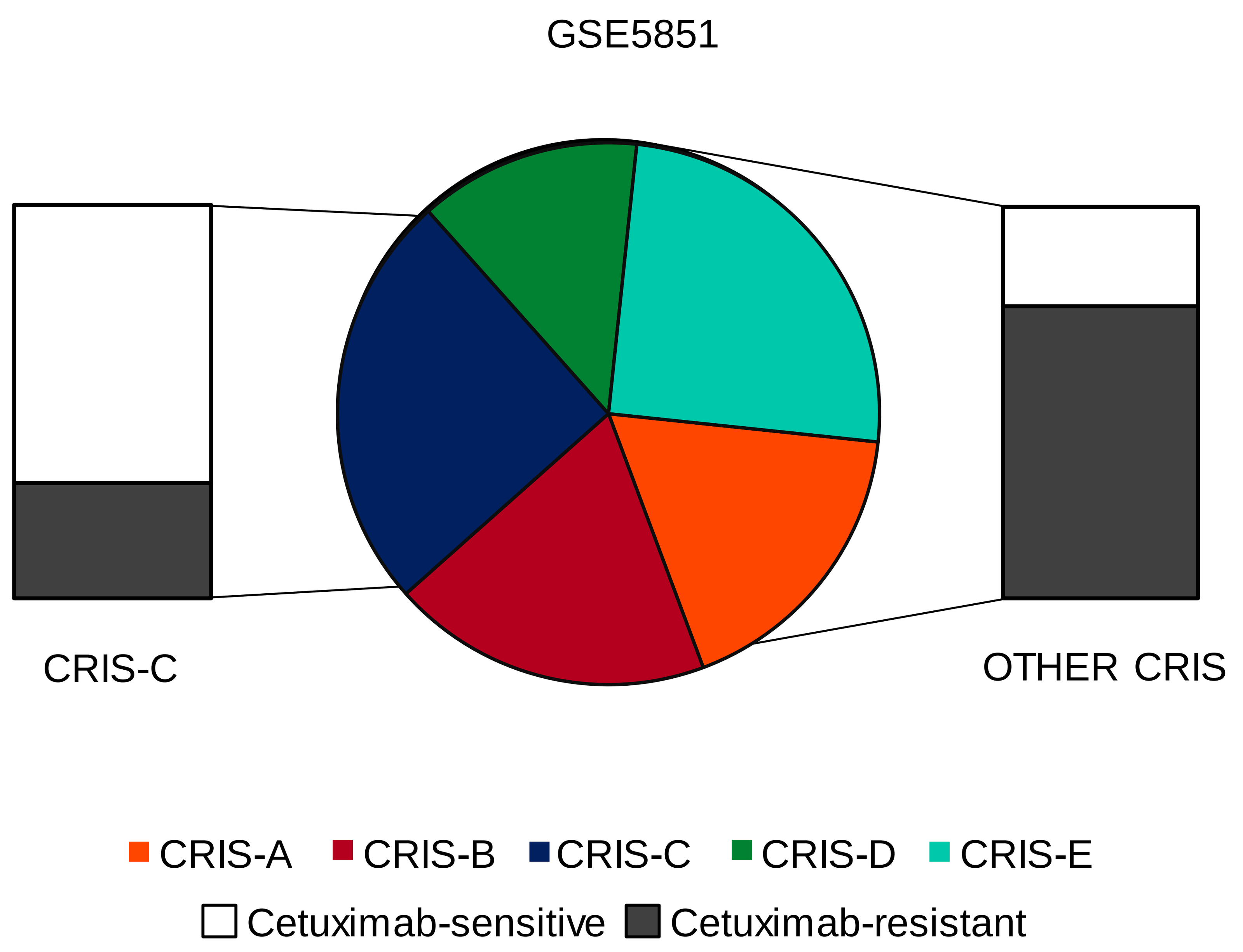

Supplementary Figure 9. Association between CRIS subtypes and sensitivity to anti-EGFR therapy in a dataset of CRC liver metastases clinically annotated for response to cetuximab.
Bar-of-pie chart showing the distribution of CRIS subtypes in GSE5851, a dataset of CRC liver metastases annotated for clinical response to cetuximab monotherapy (Khambata-Ford et al., *J. Clin. Oncol*., 2007) (pie). Bars denote the fraction of tumors responsive or resistant to cetuximab within CRIS-C or in all other CRIS classes.

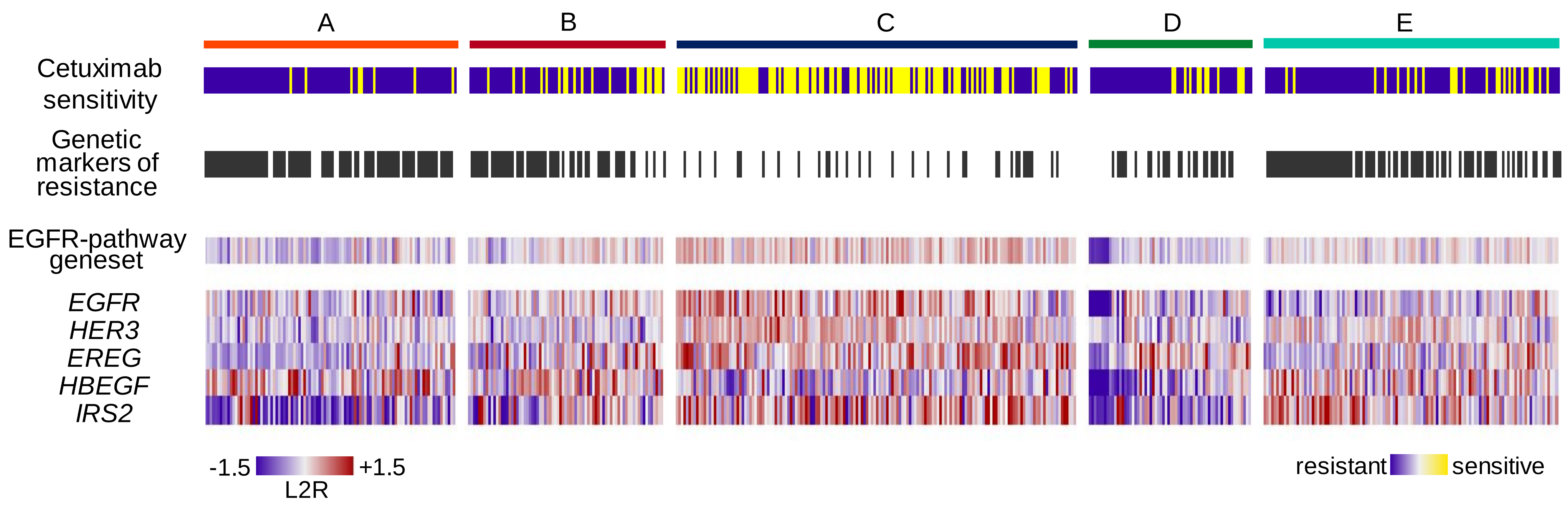

Supplementary Figure 10. Correlation between CRIS subtypes, cetuximab sensitivity, genetic predictors of resistance and expression of the EGFR-pathway activity geneset.
Heatmap showing response to cetuximab, distribution of genetic markers of resistance to cetuximab, average expression of the geneset indicative of EGFR pathway activity, and expression of individual genes of the EGFR pathway activity geneset across CRIS classes in the PDX dataset.

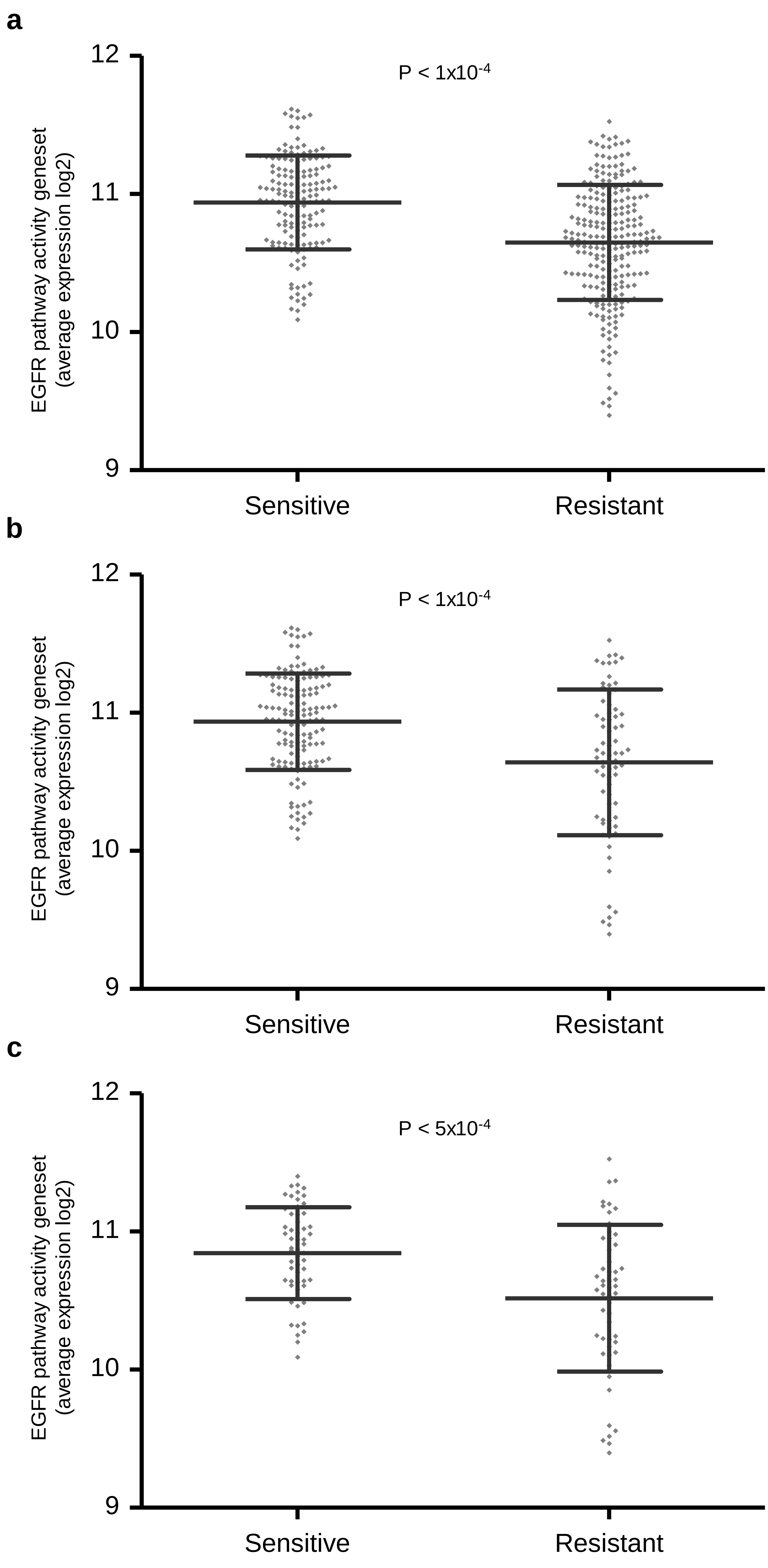

Supplementary Figure 11. Association between cetuximab sensitivity and expression of the EGFR-pathway activity geneset in the PDX dataset.
Average expression of the geneset indicative of EGFR pathway activity, according to cetuximab response (see Supplementary Figure 8), in the whole PDX dataset (**a**), in samples without known genetic causes of resistance (**b**), and in samples of (**b**) after exclusion of cases assigned to CRIS-C (**c**). Error bars indicate s.d. from the mean; P values were calculated by Wilcoxon rank sum test.

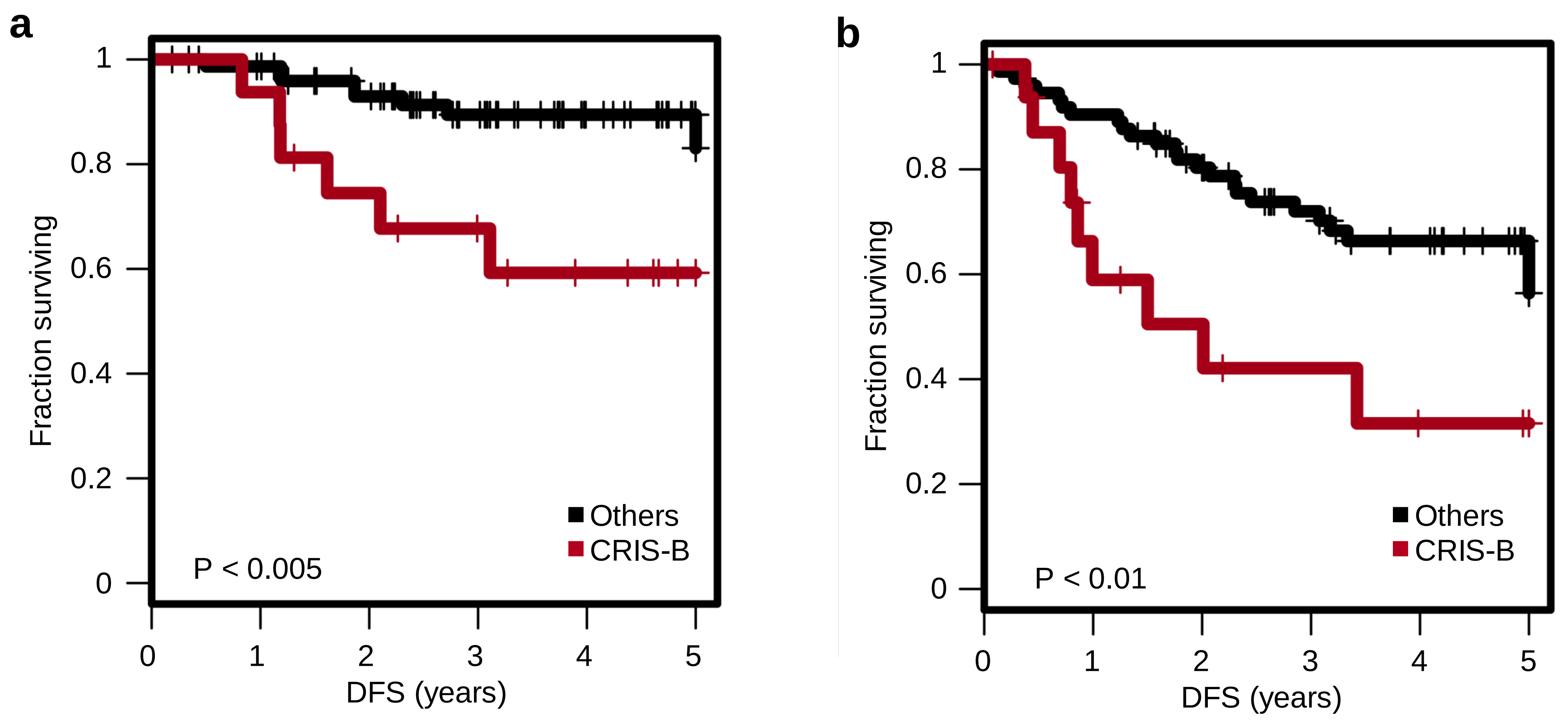

Supplementary Figure 12. Prognostic value of CRIS-B in tumors stratified by Duke's stage.
Kaplan-Meier plot comparing the disease-free survival of CRIS-B cases versus that of all other patients in Duke's stage B (**a**) and Duke Stage C (**b**) patients extracted from GSE14333. P values were calculated by log rank chi square.

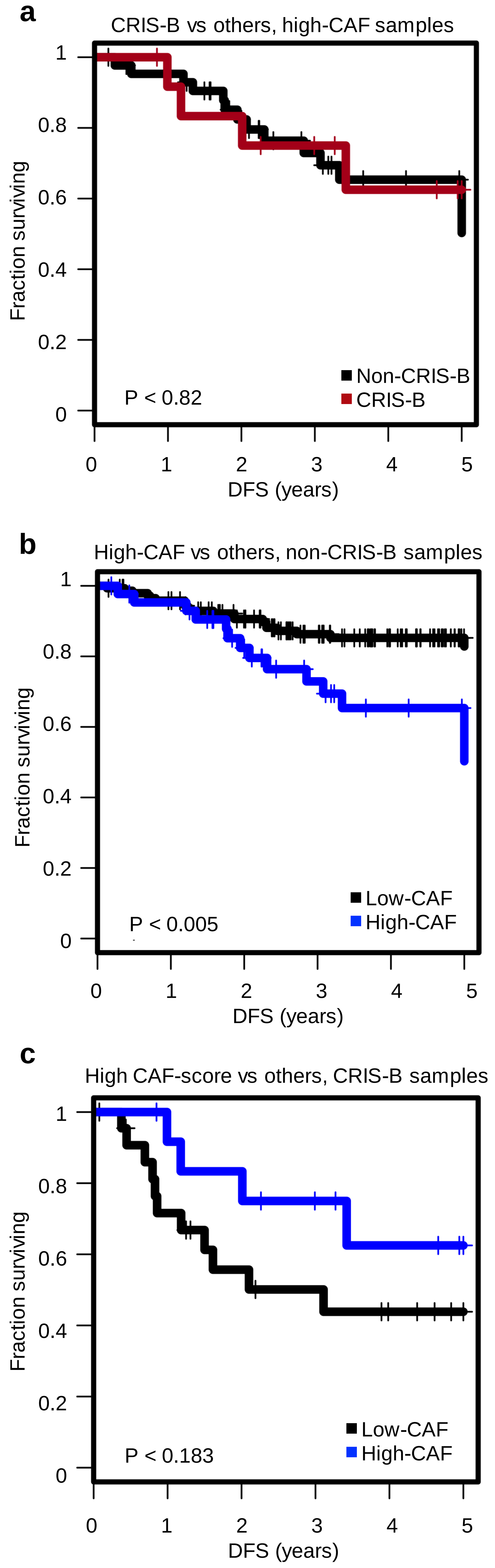

Supplementary Figure 13. Prognostic interaction between CAF infiltration and CRIS-B.
Kaplan-Meier plots comparing the disease-free survival of (**a**) CRIS-B samples with high CAF infiltration versus all other cases with high CAF infiltration; (**b**) samples with high CAF infiltration in non-CRIS-B cases versus all other non-CRIS-B cases; (**c**) CRIS-B samples with high CAF-infiltration versus all other CRIS-B samples. Data are extracted from GSE14333. P values were calculated by log-rank chi square.

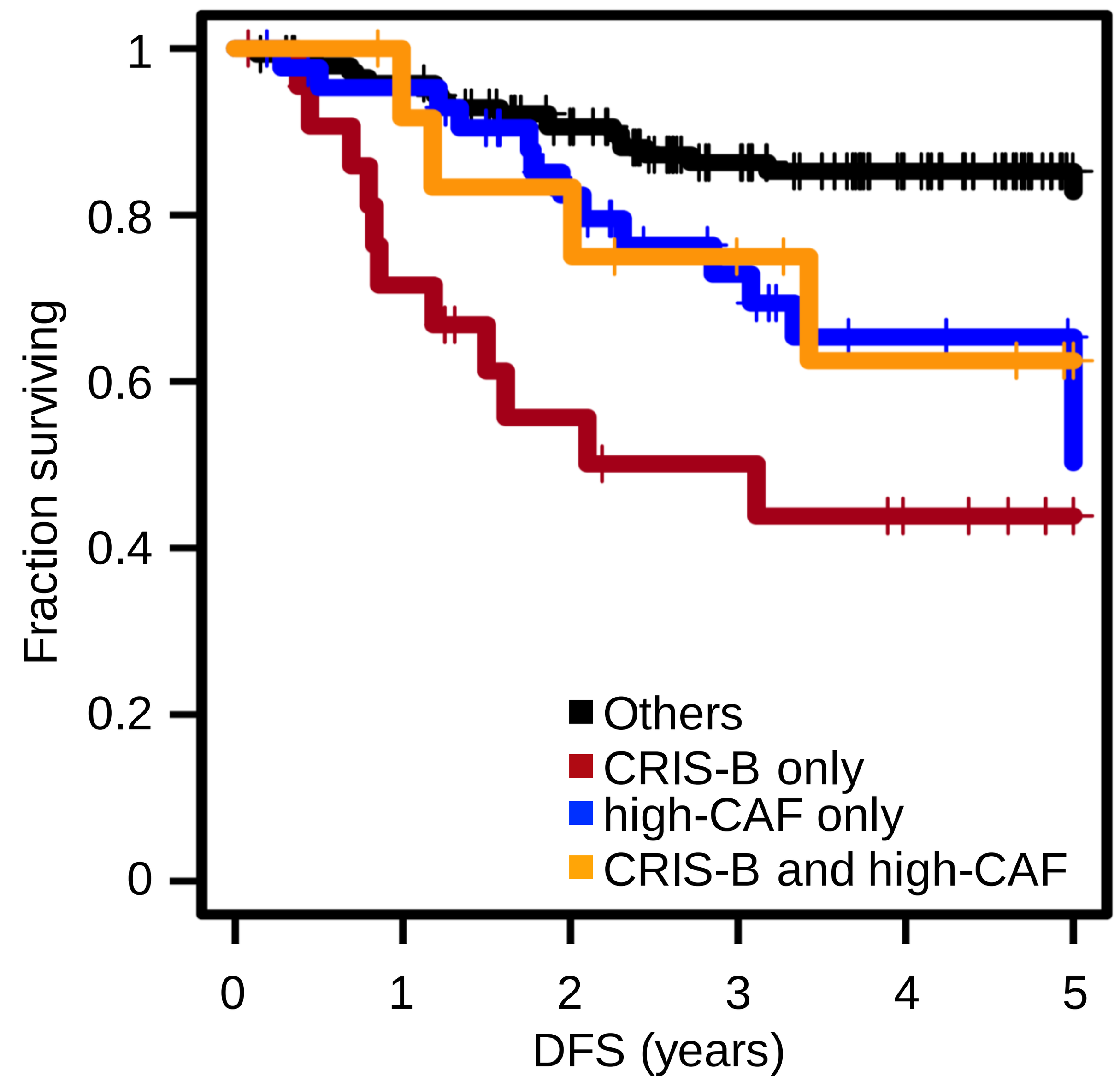

Supplementary Figure 14. Prognostic value of CRIS-B and High CAF in GSE14333.
Kaplan-Meier plot comparing the disease-free survival of CRIS-B cases with low CAF infiltration (red line), non-CRIS-B cases with high CAF infiltration (blue line) and CRIS-B cases with high CAF infiltration, versus all the other samples of the GSE14333 dataset.

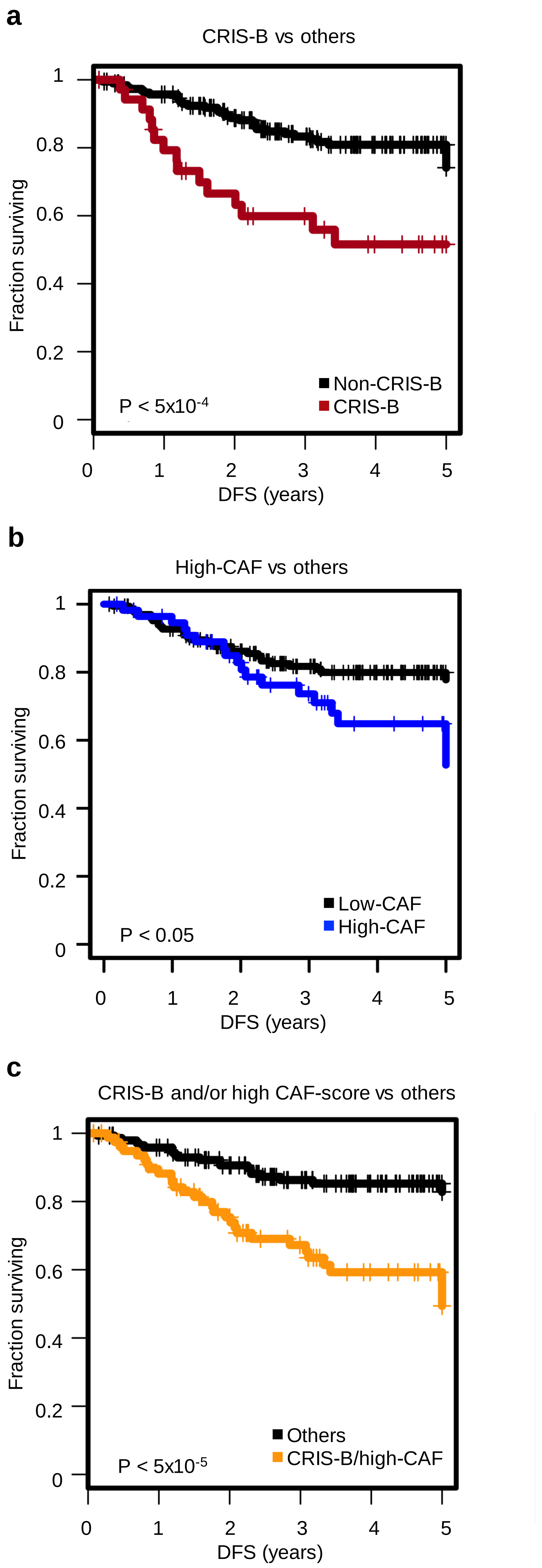

Supplementary Figure 15. Prognostic value of CRIS-B integrated with CAF score.
Kaplan-Meier plots comparing the disease-free survival of (**a**) CRIS-B samples from patients treated with adjuvant chemotherapy versus all other treated cases; (**b**) samples with high CAF infiltration versus all other cases; (**c**) CRIS-B samples or samples with high CAF infiltration versus all other samples. Data are extracted from GSE14333. P values were calculated by log-rank chi square.

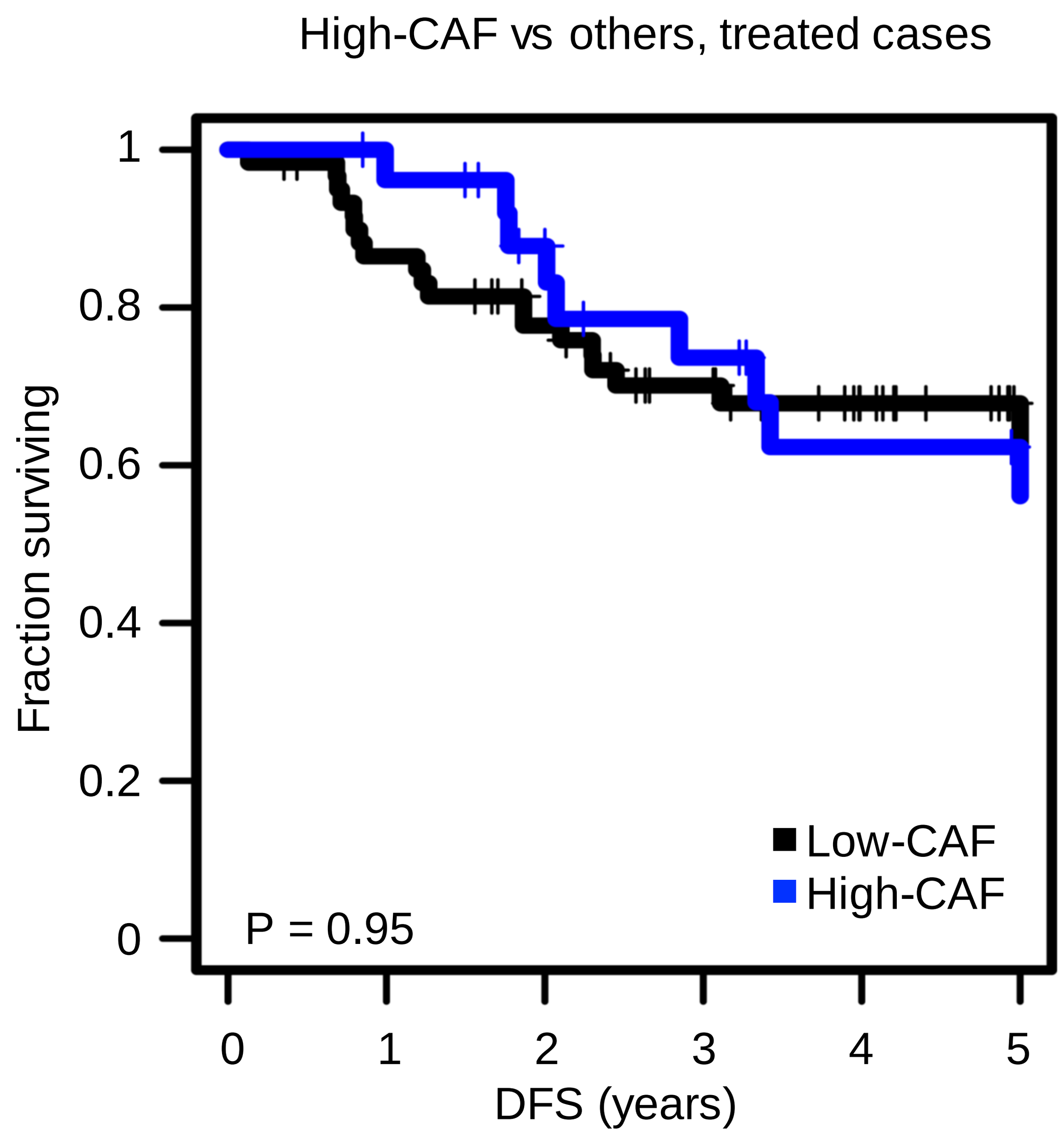

Supplementary Figure 16. Prognostic value of CAF score in cases treated with adjuvant chemotherapy. Kaplan-Meier plot comparing the disease-free survival of cases with high CAF infiltration versus that of all other cases, in patients who received adjuvant chemotherapy, extracted from GSE14333. P values were calculated by log rank chi square.

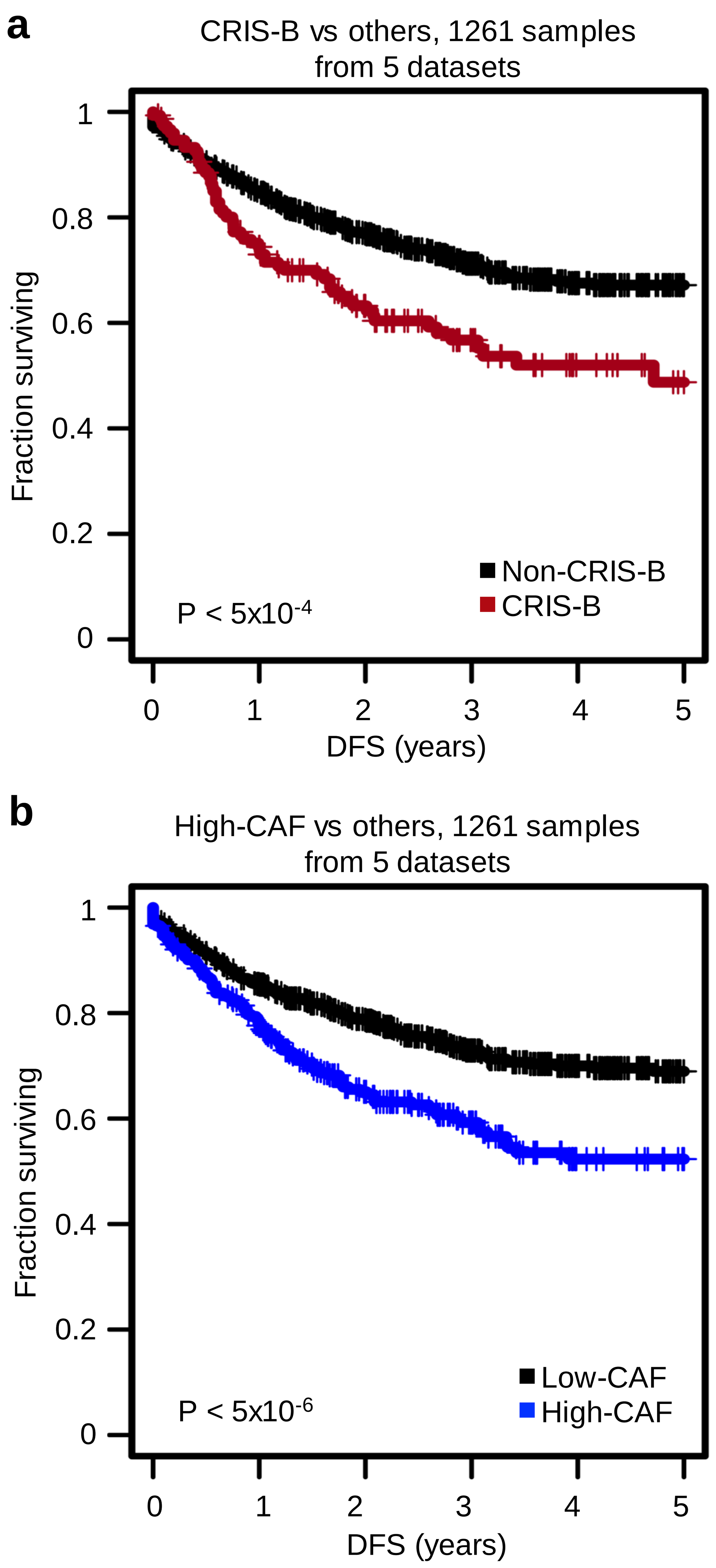

Supplementary Figure 17. Prognostic value of CAF infiltration and CRIS-B in 1261 CRC cases.
Kaplan-Meier plot comparing the disease-free survival of CRIS-B cases versus that of all other cases (**a**) or cases with high CAF infiltration versus that of all other cases (**b**), in 1261 patients collected from 5 independent datasets of primary CRC.

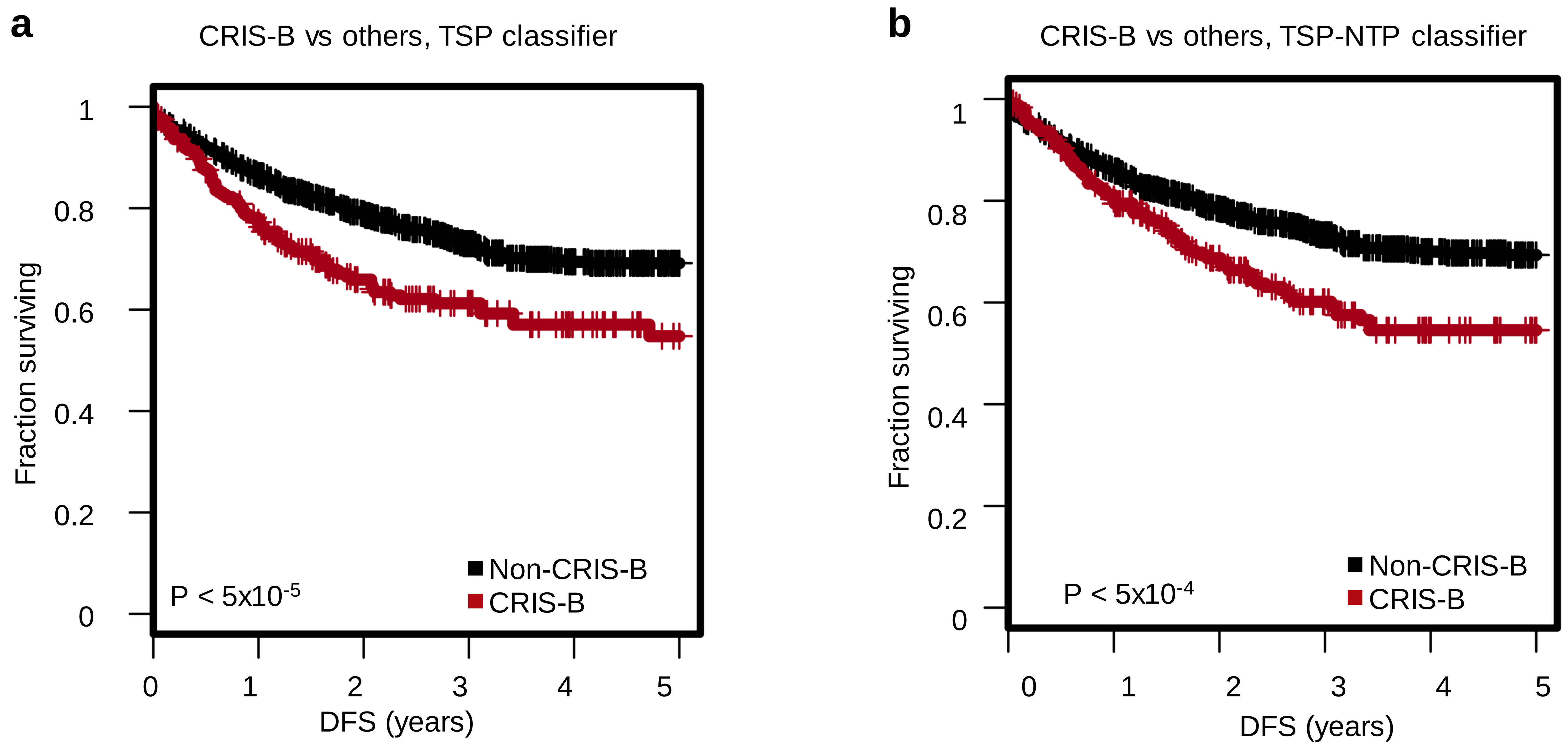

Supplementary Figure 18. Prognostic value of CRIS-TSP and CRIS-NTP80 in 1487 CRC cases.
Kaplan-Meier plots comparing the disease-free survival of CRIS-B cases versus all other cases for CRIS-TSP (**a**) or CRIS-NTP80 (**b**), in 1487 patients collected from 6 independent datasets of primary CRC.

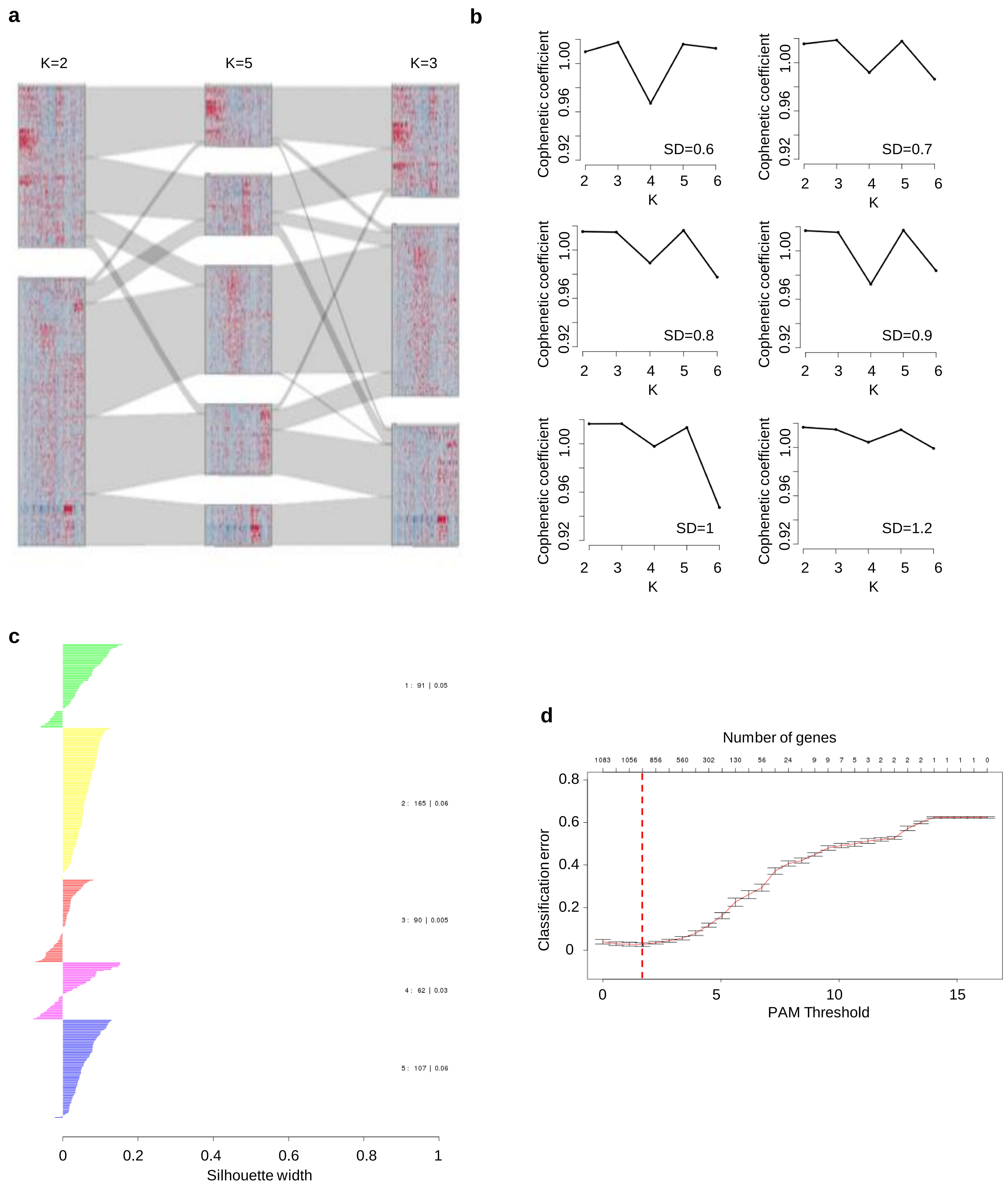

Supplementary Figure 19. Generation of the CRIS classifier.
(**a**) Caleydo view of correspondences between NMF subtypes based on different k clustering for PDX 515 dataset. (**b**) Plot of cophenetic coefficients for NMF-based consensus clustering of the PDX dataset K = 2 to K = 6 when applying different standard-deviation thresholds. (**c**) Silhouette plot for the 515 PDX CRC samples stratified by subtype. (**d**) Cross-validation of the nearest shrunken centroid from PAMR package. The dashed red vertical line indicates the optimal pam threshold of 2.

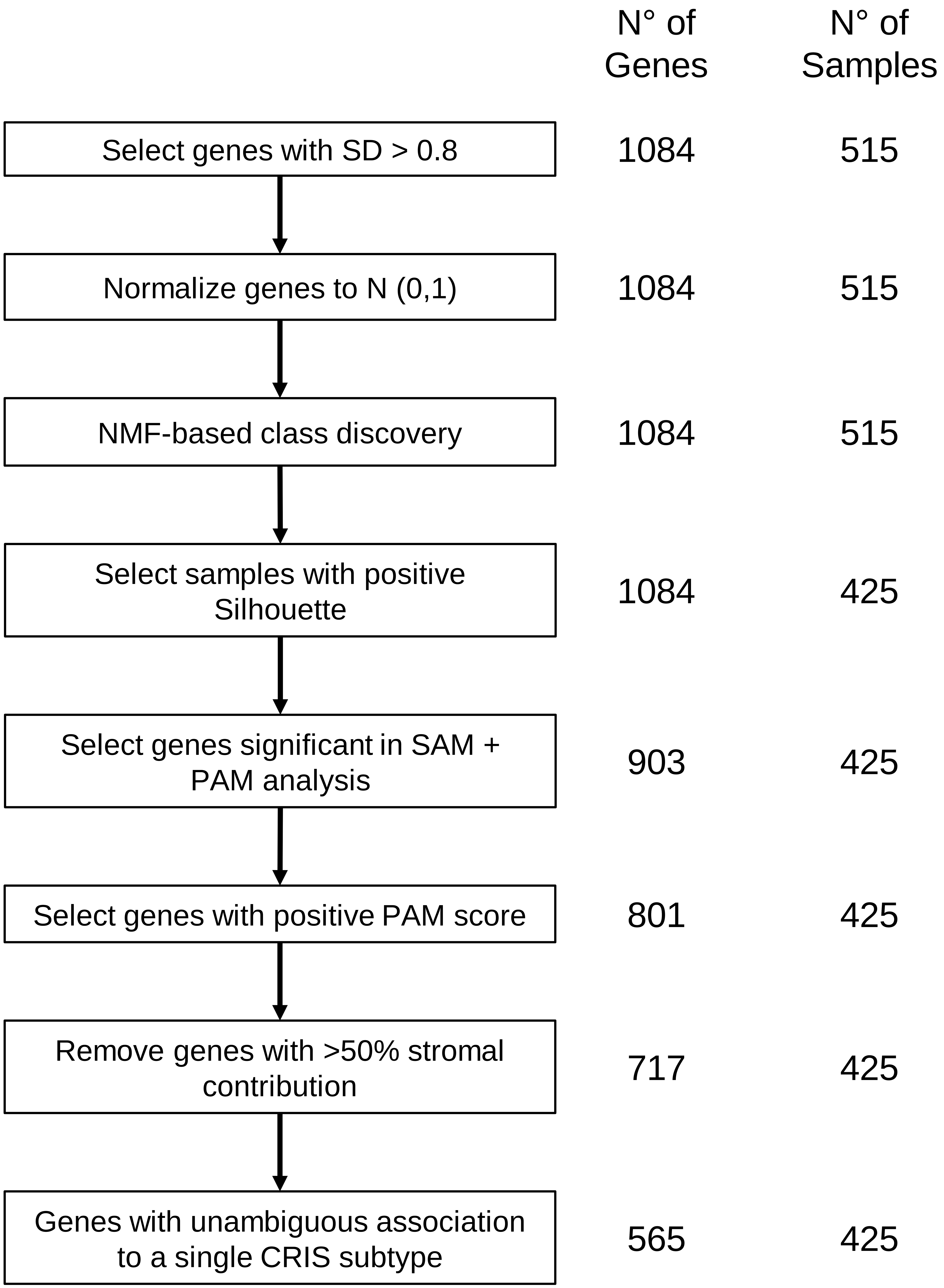

Supplementary Figure 20. Generation of the CRIS classifier.
Left column, flowchart describing the pipeline followed to identify the CRIS subtypes by NMF and to select the genes used to assign CRIS membership through the NTP algorithm. Center column, number of genes selected at each step of the pipeline. Right column, number of samples selected for the analysis.

a

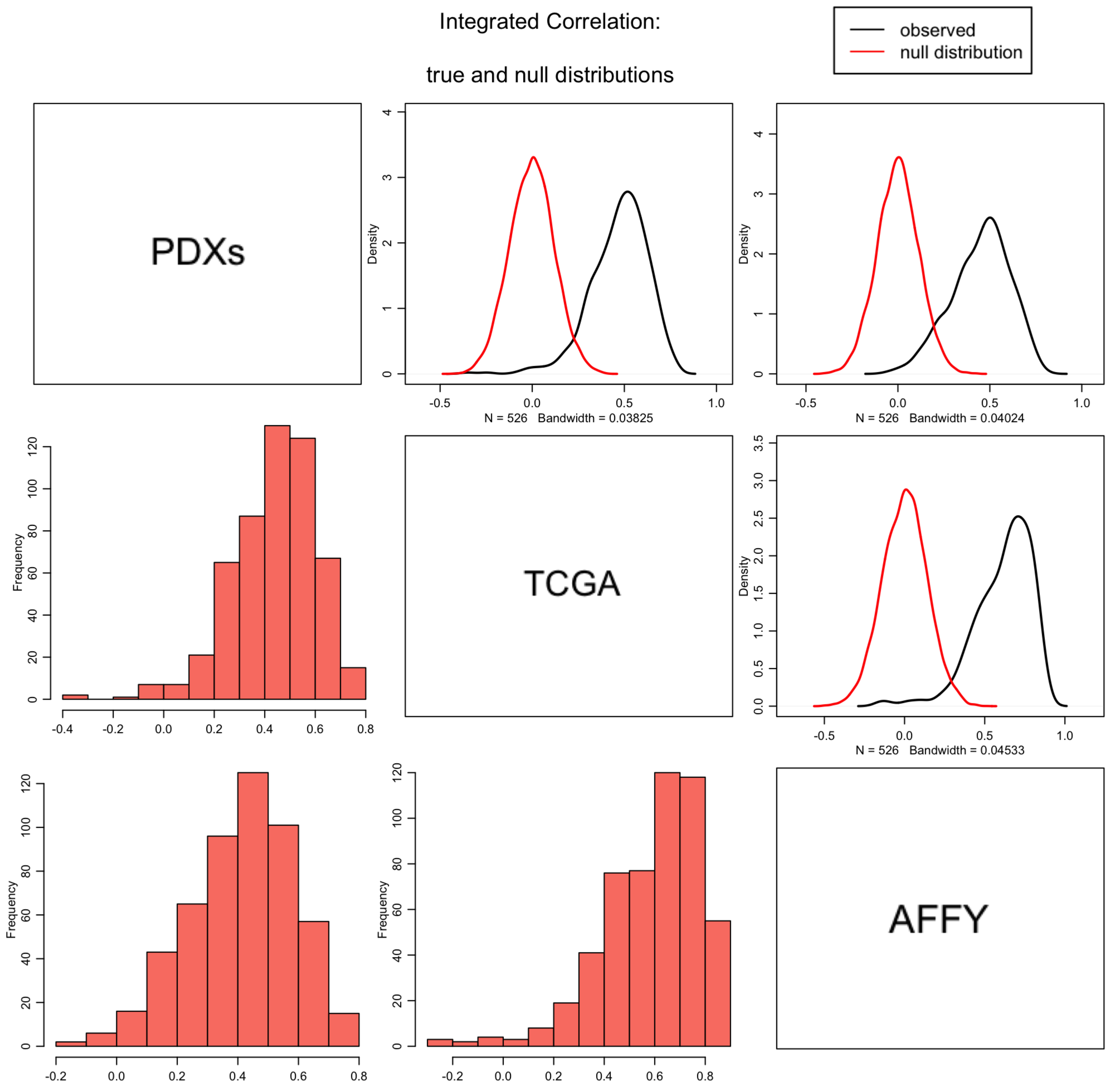

b

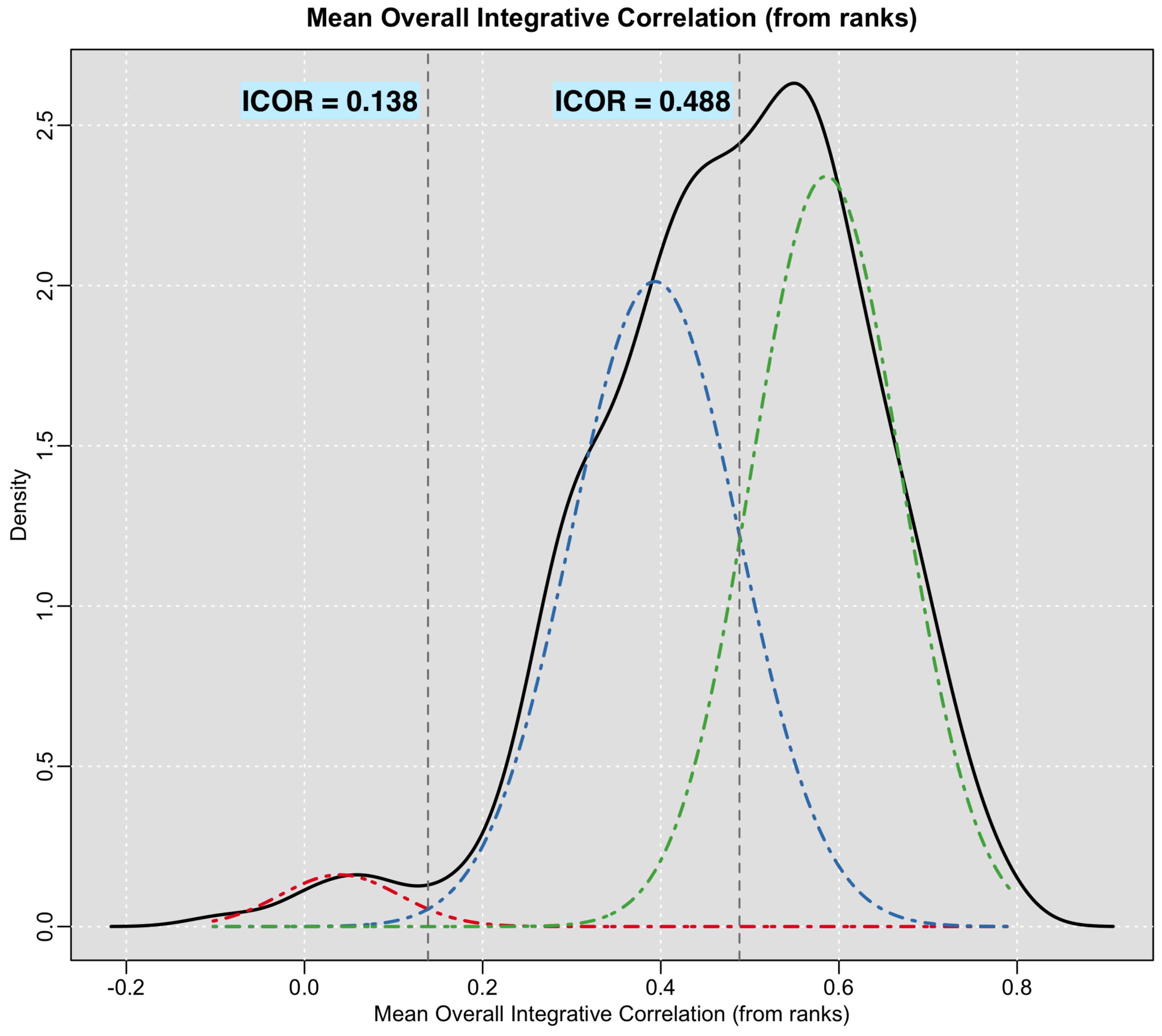

Supplementary Figure 21. Generation of the TSP CRIS.

(**a**) Histograms representing observed and the null distributions of the correlation coefficient of expression value ranks across subjects for each pair of genes across 3 datasets (1000 permutations). (**b**) Distribution of the total ICOR along with the thresholds identified by the Expectation-Maximization algorithm.

Band Amplification	CRIS Class	P value	Odds ratio
**Peak 13q12.2**	CRIS-E	4.1E-06	Infinite
**Peak 13q12.13**	CRIS-E	4.3E-06	Infinite
**Peak 11p15.5**	CRIS-D	8.8E-05	6.02
**Peak 13q22.1**	CRIS-E	1.1E-04	17.34
**Peak 7p21.3**	CRIS-C	2.9E-03	3.55
**Peak 8q12.1**	CRIS-C	7.7E-03	2.77
**Peak 8q24.21**	CRIS-C	1.1E-02	2.80
**Peak 20q12**	CRIS-C	1.4E-02	5.33
**Peak 5q22.3**	CRIS-B	1.9E-02	4.77

Supplementary Table 1. Enrichment of focal amplifications in CRIS C-D-E subtypes.
Fisher's test on focal amplification events in MSS samples from the TCGA dataset. The tests compared events in each class against the remaining MSS samples. BH correction (20% FDR) was evaluated to moderate the test.

Confusion matrix

	CRIS-A	CRIS-B	CRIS-C	CRIS-D	CRIS-E	NC
**CMS1**	36	16	0	0	2	7
**CMS2**	3	3	79	41	37	7
**CMS3**	43	3	0	1	4	2
**CMS4**	21	21	26	25	16	7
**NOLBL**	11	7	7	6	15	4

Fisher's test Odds Ratio

	CRIS-A	CRIS-B	CRIS-C	CRIS-D	CRIS-E	NC
**CMS1**	5.71	3.7	0	0	0.15	2.39
**CMS2**	0.03	0.09	6.47	2.46	1.82	0.56
**CMS3**	19.57	0.45	0	0.09	0.38	0.58
**CMS4**	0.57	2.32	0.83	1.63	0.76	1.01
**NOLBL**	0.81	1.35	0.46	0.68	2.47	1.42

Fisher's test P value
(bold = BH-FDR < 0.2; Red/blue = odds ratio greater /lower than 1)

	CRIS-A	CRIS-B	CRIS-C	CRIS-D	CRIS-E	NC
**CMS1**	0.00000	0.00027	0.00000	0.00002	0.00133	0.07499
**CMS2**	0.00000	0.00000	0.00000	0.00056	0.02523	0.22329
**CMS3**	0.00000	0.24469	0.00000	0.00111	0.07479	0.75692
**CMS4**	0.04694	0.00928	0.53393	0.07976	0.46725	1.00000
**NOLBL**	0.73018	0.47571	0.08115	0.54108	0.01335	0.52474

Supplementary Table 2: Cross-comparison of CRIS and CMS classification across 450 TCGA samples.

Deviance Residuals				
Min	1Q	Median	3Q	Max
-2.1196	-0.4497	-0.2805	0.6798	2.487
**Coefficient analysis**				
**Coefficient**	Estimate	Std. Error	t value	Pr(>|t|)
**(Intercept)**	-15.59	3.92	-3.981	6.87e-05 ***
**Genetic markers of resistance**	-2.91	0.33	-8.859	< 2e-16 ***
**EGFR pathway activity genes**	1.47	0.37	4.01	6.07e-05 ***
**CRIS-C membership**	0.49	0.15	3.278	0.00105 **
**Null deviance: 538.86 on 402 degrees of freedom**				
**Residual deviance: 333.38 on 399 degrees of freedom**				
**AIC: 341.38; Number of Fisher Scoring iterations: 5**				

Supplementary Table 3: Multiple logistic regression analysis of cetuximab sensitivity correlates in PDXs.
The "Genetic markers of resistance", "EGFR pathway activity genes" and "CRIS-C membership" were included in the analyses.

	coef	exp(coef)	se(coef)	z	p
**CRIS-B**	1.141	3.131	0.312	3.66	0.00025
**Duke's stage**	1.107	3.025	0.252	4.39	0.00001

Supplementary Table 4: Cox multiregression.
Analysis comparing the prognostic value of CRIS-B membership with that of Duke's stage in GSE14333.

Concordance between TSP and the reference CRIS classifier for different k values - training set

	K=1	K=2	K=3	K=4	K=5
**Accuracy**	0.7	0.777	0.803	0.808	0.823
**Kappa**	0.621	0.718	0.748	0.755	0.773
**Accuracy Lower**	0.653	0.73	0.759	0.765	0.782
**Accuracy Upper**	0.745	0.819	0.841	0.846	0.86
**Accuracy Null**	0.28	0.263	0.274	0.282	0.291
**Accuracy PValue**	0	0	0	0	0

Concordance between TSP and the reference CRIS classifier for different k values - internal test set

	K=1	K=2	K=3	K=4	K=5
**Accuracy**	0.556	0.718	0.714	0.77	0.746
**Kappa**	0.44	0.638	0.628	0.705	0.673
**Accuracy Lower**	0.484	0.645	0.643	0.703	0.679
**Accuracy Upper**	0.627	0.783	0.777	0.828	0.806
**Accuracy Null**	0.276	0.277	0.297	0.283	0.29
**Accuracy PValue**	0	0	0	0	0

Supplementary Table 5: Definition of the optimal K value for a CRIS TSP-based classifier.
