# Supplementary material for: Selective analysis of cancer-cell intrinsic transcriptional traits defines novel clinically relevant subtypes of colorectal cancer
Source: Nat Commun. 2017 May 31;8:15107. doi: 10.1038/ncomms15107 (PMC5499209; doi:10.1038/ncomms15107)
Supplement: Supplementary Software — The CRISclassifier, an R-Bioconductor package to classify independent gene expression datasets according to either CRIS-NTP or CRIS-TSP algorithms [file ncomms15107-s20.zip › CRISclassifier/inst/doc/cris.pdf]

CRIS: a colorectal cancer classifier based on cell autonomous gene expression

Claudio Isella, Luigi Marchionni, Enzo Medico and Andrea Bertotti

October 6, 2016

Abstract

Recent work built on published transcriptional subtypes of colorectal cancer (CRC) has shown that tumor stromal content heavily impacts CRC classification, with clinical and biological implications. Lineage-dependent transcriptional components of stromal origin could therefore dominate over more subtle, yet relevant, gene expression traits inherent to cancer cells.

Based on the notion that in patient-derived xenografts (PDXs) stromal cells of the human tumor are substituted by their murine counterparts, we assessed cancer-cell intrinsic transcriptional features by human-specific expression profiling of 515 PDXs from 244 CRC patients. We identified five CRC intrinsic subtypes (CRIS) endowed with molecular, functional and phenotypic peculiarities, many of which were not previously evidenced: (i) CRIS-A: mucinous, glycolytic, and enriched for microsatellite instability or mutations in KRAS; (ii) CRIS-B: marked TGF-Beta pathway activity, epithelial-mesenchymal transition, and poor prognosis; (iii) CRIS-C: elevated EGFR signaling and sensitivity to treatment with EGFR inhibitors; (iv) CRIS-D: WNT activation accompanied by IGF2 overexpression and amplification; (v) CRIS-E: Paneth cell-like phenotype and higher frequency of TP53 mutation.

CRIS subtypes successfully categorized independent sets of primary and metastatic CRC tumors, with limited overlap on existing transcriptional classes and unprecedented predictive and prognostic performances. Integration of cancer-cell intrinsic classification by CRIS with stroma-centered transcriptional profiles further enhanced prognostic significance.

Contents

1 Introduction **3**

2 CRIS NTP classifier **3**

3 CRIS TSP classifier **4**

3.1 Classifying samples using the kTSO set 6

4 System Information **6**

5 References **7**

1 Introduction

A number of classification systems based on gene expression have been proposed that stratify colorectal cancer (CRC) in subgroups with distinct molecular and clinical features[1, 2, 3, 4, 5, 6, 7]. These classification efforts have been recently consolidated by a multi-institutional initiative that comprehensively cross-compared the different subtype assignments on a common set of samples, leading to the definition of the Consensus Molecular Subtypes (CMS)[10].

Interestingly, we and others independently reported that a large portion of the genes sustaining the SSM subtype (CMS4 within the consensus molecular subtypes) are of stromal origin[8, 9].

Likelly, in whole tumor lysates the transcriptional consequences of biologically meaningful traits that are inherent to cancer cells might be obscured by the presence of a dominant, lineage-dependent transcriptional component of stromal origin.

To tackle this issue, we exploited a large collection (n = 515 samples from 244 patients) of patient-derived xenografts (PDXs), in which the stromal components of the original tumor are substituted by their murine counterparts so that detection of their transcripts can be avoided by appropriate use of human-specific arrays. By doing so, we defined the CRIS subtypes and generated an NTP based classifier [11]. This package is ment to provide feasible access to the CRIS classification of CRC gene expression profile. The classifier will subdivide colorectal cancer in 5 distinct subtypes on the basis of 5 five centroids, collectivelly defined by 566 unique gene symbols.

The package is loaded with the command

2 CRIS NTP classifier

```
> library(CRISclassifier)
```

In the first example we illustrate how to classify colorectal cancer gene expression profiles according to CRIS. In the example we are employ a demo dataset of 38 cancer cell lines. The function requires the gene expression data matrix in linear format defined as .gct file, and the first colmun annotated to gene Symbol.

```
> demo <- list.files(pattern="txt.gz$", system.file("data",package="CRISclassifier"), full
> print(demo)

[1] "/private/var/folders/5h/rj1zvwhj20sglw4pg7r7_62m0000gn/T/RtmpMaXxaU/Rinst5f7f3e47ae95

> cris_classifier(input.exp.filename = demo, output.name="cris", nresmpl=1)

[1] 38
[1] "sample # 1"
[1] "sample # 2"
[1] "sample # 3"
[1] "sample # 4"
[1] "sample # 5"
[1] "sample # 6"
[1] "sample # 7"
```

```
[1] "sample # 8"
[1] "sample # 9"
[1] "sample # 10"
[1] "sample # 11"
[1] "sample # 12"
[1] "sample # 13"
[1] "sample # 14"
[1] "sample # 15"
[1] "sample # 16"
[1] "sample # 17"
[1] "sample # 18"
[1] "sample # 19"
[1] "sample # 20"
[1] "sample # 21"
[1] "sample # 22"
[1] "sample # 23"
[1] "sample # 24"
[1] "sample # 25"
[1] "sample # 26"
[1] "sample # 27"
[1] "sample # 28"
[1] "sample # 29"
[1] "sample # 30"
[1] "sample # 31"
[1] "sample # 32"
[1] "sample # 33"
[1] "sample # 34"
[1] "sample # 35"
[1] "sample # 36"
[1] "sample # 37"
[1] "sample # 38"
null device
          1

>
```

The function will generate a default output in the file-system: CRIS_prediction_result.xls: Prediction result for the input dataset CRIS_features.xls: List of marker genes mapped in the dataset CRIS_heatmap.png: Heatmap of marker genes in the dataset CRIS_FDR_sample_bar.png: Predicted sample labels at FDR_0.2_CRIS_FDR.png Plot of FDR CRIS_heatmap_legend.png Color map for SD -3 - +3.

3 CRIS TSP classifier

The *CRISclassifier* package also allows to predict CRIS classes based on gene expression using a classifier based on kTSP [14, 13, 12].

THe *predictCRISclassKTSP* function allows to predic CRIS classes using a pre-defined kTSP set. This function accepts a numeric matrix contining gene expresion values as its only argument. The rownames of this gene expression data must containg valid gene symbols corresponding to the used by the classifiers.

The function can handle missing genes, provided that the remaning pais allow to perform all 10 pairwise comparisons among the five CRIS classes (CRISA, CRISB, CRISC, CRISD, and CRISE)

Load the library.

```
> require(predictCRIS)
```

Load the example data contained in the *predictCRIS* package.

```
> data(matList)
> data(phenoList)
```

The object *matList* is a list of matrices containing differential gene expression data from 2 distinct dataset. The first matrix accounts for all CRISS genes used by the kTSP classifier (80 genes), while the second matrix only accounts for 72 of the 80 genes. analysis resulst from three distinct experiments. The object *phenoList* contains the corresponding CRIS classes obtained by using all 526 CRIS genes and the Nearest Template Predictor (NTP). Below is shown the structure of these objects:

```
> sapply(matList, class)

Training  Testing
"matrix" "matrix"

> sapply(matList, dim)

     Training Testing
[1,]       80      72
[2,]      416     208

> sapply(phenoList, class)

Training  Testing
"factor" "factor"

> sapply(phenoList, length)

Training  Testing
     416      208

> sapply(phenoList, summary)

      Training Testing
CRISA       98      50
CRISB       61      31
CRISC      117      58
CRISD       68      34
CRISE       72      35
```

3.1 Classifying samples using the kTSO set

To classify new samples using the kTSP classifiers one can use the *predictCRISclassK-TSP* as follows (for one dataset):

```
> ### Valid gene expression matrix with all CRIS genes
> newMat <- matList$Training
> ### To make predictions on 1 matrix
> newPreds <- predictCRISclassKTSP(newMat)
> ### Counts classifications
> summary(newPreds)

                      Length Class  Mode
tspSetClassPercent    2080   -none- numeric
tspSetClassPredsFinal  416   factor numeric

> ### NPT classification
> refClass <- phenoList$Training
```

To classify new samples using the kTSP classifiers one can use the *predictCRISclassKTSP* as follows (for multiple datasets):

```
> ### For all matrices
> newPredsList <- lapply(matList, predictCRISclassKTSP)
> ### Count classifications
> lapply(newPredsList, summary)

$Training
                      Length Class  Mode
tspSetClassPercent    2080   -none- numeric
tspSetClassPredsFinal  416   factor numeric

$Testing
                      Length Class  Mode
tspSetClassPercent    1040   -none- numeric
tspSetClassPredsFinal  208   factor numeric
```

4 System Information

Session information:

```
> toLatex(sessionInfo())

\begin{itemize}\raggedright
  \item R version 3.3.1 (2016-06-21), \verb|x86_64-apple-darwin13.4.0|
  \item Locale: \verb|C/en_GB.UTF-8/en_GB.UTF-8/C/en_GB.UTF-8/en_GB.UTF-8|
  \item Base packages: base, datasets, grDevices, graphics, methods,
    stats, utils
  \item Other packages: CRISclassifier~0.99.0
  \item Loaded via a namespace (and not attached): tools~3.3.1
\end{itemize}
```

5 References

References

[1] Sadanandam, A. et al. *A colorectal cancer classification system that associates cellular phenotype and responses to therapy* Nat Med 19, 619-25 (2013)

[2] De Sousa E Melo, F. et al. *Poor-prognosis colon cancer is defined by a molecularly distinct subtype and develops from serrated precursor lesions.* Nat Med 19, 614-8 (2013)

[3] Marisa, L. et al. *Gene expression classification of colon cancer into molecular subtypes: characterization, validation, and prognostic value.* PLoS Med 10, e1001453 (2013)

[4] Roepman, P. et al. *Colorectal cancer intrinsic subtypes predict chemotherapy benefit, deficient mismatch repair and epithelial-to-mesenchymal transition.* Int J Cancer 134, 552-62 (2014).

[5] Budinska, E. et al. *Gene expression patterns unveil a new level of molecular heterogeneity in colorectal cancer.* J Pathol 231, 63-76 (2013)

[6] Schlicker, A. et al. *Subtypes of primary colorectal tumors correlate with response to targeted treatment in colorectal cell lines.* BMC Med Genomics 5, 66 (2012)

[7] Perez-Villamil, B. et al. *Colon cancer molecular subtypes identified by expression profiling and associated to stroma, mucinous type and different clinical behavior.* BMC Cancer 12, 260 (2012)

[8] Isella, C. et al. *Stromal contribution to the colorectal cancer transcriptome.* Nat Genet (2015)

[9] Calon, A. et al. *Stromal gene expression defines poor-prognosis subtypes in colorectal cancer.* Nat Genet (2015)

[10] Guinney, J. et al. *The consensus molecular subtypes of colorectal cancer.* Nat Med (2015)

[11] Hoshida, Y. *Nearest Template Prediction: A Single-Sample-Based Flexible Class Prediction with Confidence Assessment* PlosOne Nov. (2010) http://dx.doi.org/10.1371/journal.pone.0015543

[12] Tan, A et al. *Simple decision rules for classifying human cancers from gene expression profiles* Bioinformatics (2005)

[13] Geman, A et al. *Classifying gene expression profiles from pairwise mRNA comparisons* Stat Appl Genet Mol Biol (204)

[14] Marchionni, L et al. 2013 *Breast Neoplasms; Cohort Studies; Computational Biology; Gene Expression Profiling; Humans; Prognosis; Reproducibility of Results; Software* BMC Genomics 2013
